# Remdesivir and corticosteroids in the treatment of hospitalized COVID-19 patients

**DOI:** 10.1038/s41598-023-31544-5

**Published:** 2023-03-18

**Authors:** Luís Coelho, Fatima Falcão, Pedro Póvoa, Erica Viegas, Antonio Pais Martins, Eduarda Carmo, Candida Fonseca, Luis Campos, Kamal Mansinho, Inês Carmo, Joana Soares, Mariana Solano, Dina Mendes, Ana Cláudia Miranda, Antonio Carvalho, Ana Mirco, Helena Farinha, Isabel Aldir, José Correia

**Affiliations:** 1 Polyvalent Intensive Care Unit, Hospital de São Francisco Xavier, Centro Hospitalar de Lisboa Ocidental, Lisbon, Portugal; 2 NOVA Medical School, CHRC, New University of Lisbon, Lisbon, Portugal; 3 Pharmacy Department, Centro Hospitalar de Lisboa Ocidental, Lisbon, Portugal; 4 Faculty of Pharmacy of the University of Lisbon, Lisbon, Portugal; 5 Center for Clinical Epidemiology and Research Unit of Clinical Epidemiology, OUH Odense University Hospital, Odense, Denmark; 6 Surgical Intensive Care Unit, Hospital de S. Francisco Xavier, Centro Hospitalar de Lisboa Ocidental, Lisbon, Portugal; 7 Polyvalent Intensive Care Unit, Hospital de Egas Moniz, Centro Hospitalar de Lisboa Ocidental, Lisbon, Portugal; 8 Internal Medicine, Hospital de S. Francisco Xavier, Centro Hospitalar de Lisboa Ocidental, Lisbon, Portugal; 9 Infecciology, Hospital de Egas Moniz, Centro Hospitalar de Lisboa Ocidental, Lisbon, Portugal; 10 Pharmacy and Therapeutics Committee, Centro Hospitalar de Lisboa Ocidental, Lisbon, Portugal

**Keywords:** Diseases, Medical research, Drug safety, Pharmaceutics, Pharmacology

## Abstract

Coronavirus disease 2019 (COVID-19) is a pandemic infection caused by the newly discovered severe acute respiratory syndrome coronavirus 2. Remdesivir (RDV) and corticosteroids are used mainly in COVID-19 patients with acute respiratory failure. The main objective of the study was to assess the effectiveness of remdesivir with and without corticosteroids in the treatment of COVID-19 patients. We conducted a prospective observational study, including adult patients consecutively hospitalized with confirmed COVID-19 and acute respiratory failure. Patients were divided according to treatment strategy: RDV alone versus RDV with corticosteroids. The primary outcome was the time to recovery in both treatment groups. We included 374 COVID-19 adult patients, 184 were treated with RDV, and 190 were treated with RDV and corticosteroid. Patients in the RDV group had a shorter time to recovery in comparison with patients in the RDV plus corticosteroids group at 28 days after admission [11 vs. 16 days (95% confidence Interval 9.7–12.8; 14.9–17.1; *p* = .016)]. Patients treated with RDV alone had a shorter length of hospital stay. The use of corticosteroids as adjunctive therapy of RDV was not associated with improvement in mortality of COVID-19 patients.

## Introduction

Coronavirus disease 2019 (COVID-19) is a pandemic infection caused by the newly discovered severe acute respiratory syndrome coronavirus 2 (SARS-CoV2). The first cases of the disease emerged in Wuhan, China, in December 2019 and quickly spread across the globe infecting millions of people and causing more than 5 million deaths^1^.

Since COVID-19 was declared a global pandemic, several treatments have been tested, but so far few have been shown to have significantly decreased the mortality of COVID-19 patients. Currently, supportive care measures such as mechanical ventilation, oxygenation, and fluid management remain the standard of care^2^.

Several classes of drugs have been tested, particularly in patients with moderate to severe infection, including several antiviral agents. One of these agents is remdesivir (RDV), an RNA-dependent polymerase inhibitor, developed in 2017 as a treatment for Ebola virus infection^3^. In July 2020, European Medicines Agency (EMA) granted this antiviral a conditional marketing authorisation for COVID-19 treatment. RDV efficacy and safety profile were evaluated in NIAID ACTT-1, a randomized double blind placebo controlled trial and further scrutinized in the ACTT-2 and ACTT-3^3–5^. The clinical benefit of remdesivir was most apparent in patients receiving oxygen, and not on invasive mechanical ventilation at Day 1.

Immunomodulatory drugs, such as corticosteroids, to control the hyperinflammatory response frequently seen in COVID-19 infection, have also been studied. So far, some studies have shown that the use of corticosteroids could reduce the risk of 28-day mortality, namely in patients under mechanical ventilation^6^.

The main objective of this study was to assess the effectiveness of remdesivir with and without corticosteroids in the treatment of COVID-19 patients in a Portuguese Hospital Centre.

## Methods

### Study setting and design

We conducted a prospective observational cohort study of COVID-19 patients treated with RDV alone or with the association of RDV plus corticosteroids, collecting data since RDV’s approval, from June 2020 to December 2020. Eligible patients included adults consecutively hospitalized with confirmed SARS-CoV-2 infection and acute respiratory. Patients included in the RDV plus corticosteroids group were treated with dexamethasone 6 mg/day for 10 days, or until discharge or death.

### Ethical declarations

The study protocol was approved by the institutional review board of Centro Hospitalar Lisboa Ocidental and conducted in accordance with Good Clinical Practice guidelines and the principles of the Declaration of Helsinki. All of the authors vouched for the accuracy and completeness of the data. The informed consent was waived by the ethical committee since the study was observational and there was no diagnostic or therapeutic intervention outside the usual clinical practice or the need for additional collection of biological samples.

### Data collection

We obtained data through our institution’s electronic medical records (EMRs).

### Primary endpoint

The primary endpoint was the time to recovery in both treatment groups. Clinical status was assessed at 28 days to evaluate effectiveness of drug treatments. Clinical recovery on D28 was defined as a decrease of up to 1 or 2 points on the ordinal scale or live discharge was also evaluated.

### Secondary endpoints

Secondary endpoints were the cumulative mortality through day 28, the scores on the World Health Organization (WHO) ordinal scale (OS) of disease severity at day 28, the status of being alive and not using mechanical ventilation or extracorporeal membrane oxygenation (ECMO) at day 28, and not being hospitalized at day 28. The incidence of adverse events was monitored and assessed.

### Statistical analysis

All data are expressed as mean to Gaussian distributed variables and as medians [with interquartile range (IQR)] to non-normally distributed variables unless specified otherwise. Comparisons between groups were performed with unpaired Student's t-test, one-way ANOVA, Mann–Whitney U or Kruskal–Wallis H-tests for continuous variables according to data distribution. Chi-square tests were used to carry out comparisons between categorical variables. Linear logistic regression modelling for time to recovery was carried. Kaplan–Meier survival curve and log-rank test were also obtained to ascertain and compare survival between groups. Cox proportional hazards regression analysis was performed with adjustment for comorbidities or severity of patients evaluated by WHO Ordinal Scale. A *p* value of less than 0.05 was considered for statistical significance and usual confidence intervals of 95% were chosen. Statistical analysis was performed using the SPSS software version 26.0 (Supplementary Information 1).

## Results

### Patients and treatment

**Figure 1:**
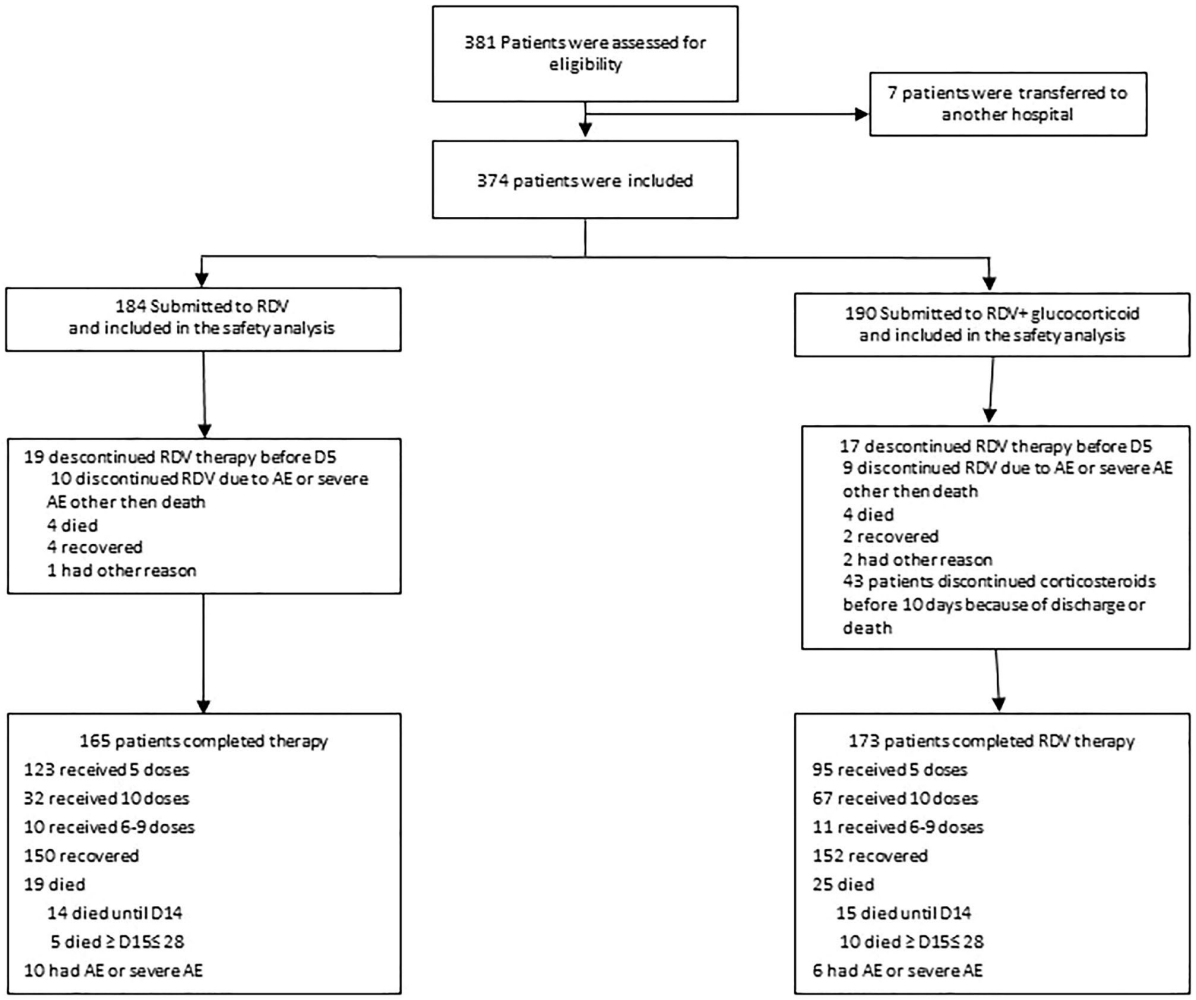
Patient distribution.

Of the 374 included patients, 184 were treated with RDV and 190 were treated with RDV and corticosteroid (Fig. 1). Our population included 298 patients with moderate disease (ordinal score of 5, requiring supplemental oxygen) and 76 with severe disease (ordinal score of 6 or 7, receiving non-invasive or invasive ventilation).

The mean age of the patients was 67.3 years, 67.9% were male, 64.3% were obese or overweight, and the median time from symptoms to RDV therapy was 7 days. One or more coexisting conditions were present in 75.1% of the patients. Patients in the group of RDV plus corticosteroids presented a significant higher severity in the evaluation OS (*p* = 0.016). The overall hospital mortality of COVID-19 patients was 13.9% (Table 1). No significant differences were observed between comorbidities in both groups (Table 2).

**Table 1:** Demographic and clinical characteristics of the patients at baseline.

Characteristics	All Patients	RDV	RDV + CORT	p
	n = 374	n = 184	n = 190	
Age				
Mean (years ± SD)	67.3 ± 15.3	65.8 ± 15.2	68.8 ± 15.3	.59
Distribution (n; %)				.14
< 40	16 (4.3)	10 (5.4)	6 (3.2)	
40—64	127 (34.0)	71 (38.6)	56 (29.5)	
≥ 65	231 (61.7)	103 (56.0)	128 (67.4)	
Sex (n; %)				.65
Female	120 (32.1)	57 (31.0)	63 (33.2)	
Male	254 (67.9)	127 (69.0)	127 (66.8)	
Body-mass index (n/total; %)				.1
Normal/Underweight (< 25)	106/297 (35.7)	61/152 (40.1)	45/145 (31.0)	
Obese/Overweight (≥ 25)	191/297 (64.3)	91/152 (59.9)	100/145 (69.0)	
Time from symptom onset to RDV (days; SD)	n = 354	n = 173	n = 181	
Mean	7.2 ± 4.2	7.0 ± 3.9	7.3 ± 4.4	.1
Length of Therapy (days; SD)				
Mean	6.2 ± 2.5	5.8 ± 2.2	6.7 ± 2.6	< .001
Score on ordinal scale—(n; %)				.016
5	298 (79.7)	158 (85.9)	140 (73.7)	
6	25 (6.7)	6 (3.3)	19 (10)	
7	51 (13.6)	20 (10.9)	31 (16.3)	
Mortality (n; %)	52 (13.9)	23 (12.5)	29 (15.2)	.459

**Table 2:** Comorbidity distribution of COVID-19 patients.

Comorbidities	All patients	RDV	RDV + CORT	*p*
Arterial hypertension	233	112	121	.59
Coronary heart disease	38	15	23	.23
Chronic heart failure	18	9	9	1
Diabetes	134	57	67	.38
COPD	24	10	14	.38
Asthma	17	11	6	.32
Chronic renal disease	17	8	9	1
Chronic hepatic disease	8	2	5	.33
Oncologic disease	46	23	23	1
HIV infection	3	2	1	.61

*COPD* Chronic obstrutive pulmonary disease, *HIV* human immunodeficiency virus.

### Primary endpoint

Patients in the RDV group had a shorter time to recovery in comparison with patients in the RDV plus corticosteroids group at 28 days after admission [11 vs. 16 days (95% confidence Interval (CI) 9.7–12.8; 14.9–17.1; *p* = 0.016)].

Also, hospital free days at 28 days were higher in the RDV group [13.8 vs. 11.5 days, (95% CI 12.5–15; 10.3–12.6; *p* = 0.001)]. Overall, the length of hospital stay of patients treated with RDV was shorter than that of patients in the RDV plus corticosteroids group [17.6 vs 21.7 days; (95% CI 15.1–20.1; 18.6–24.8; *p* = 0.001)].

### Secondary endpoints

**Figure 2:**
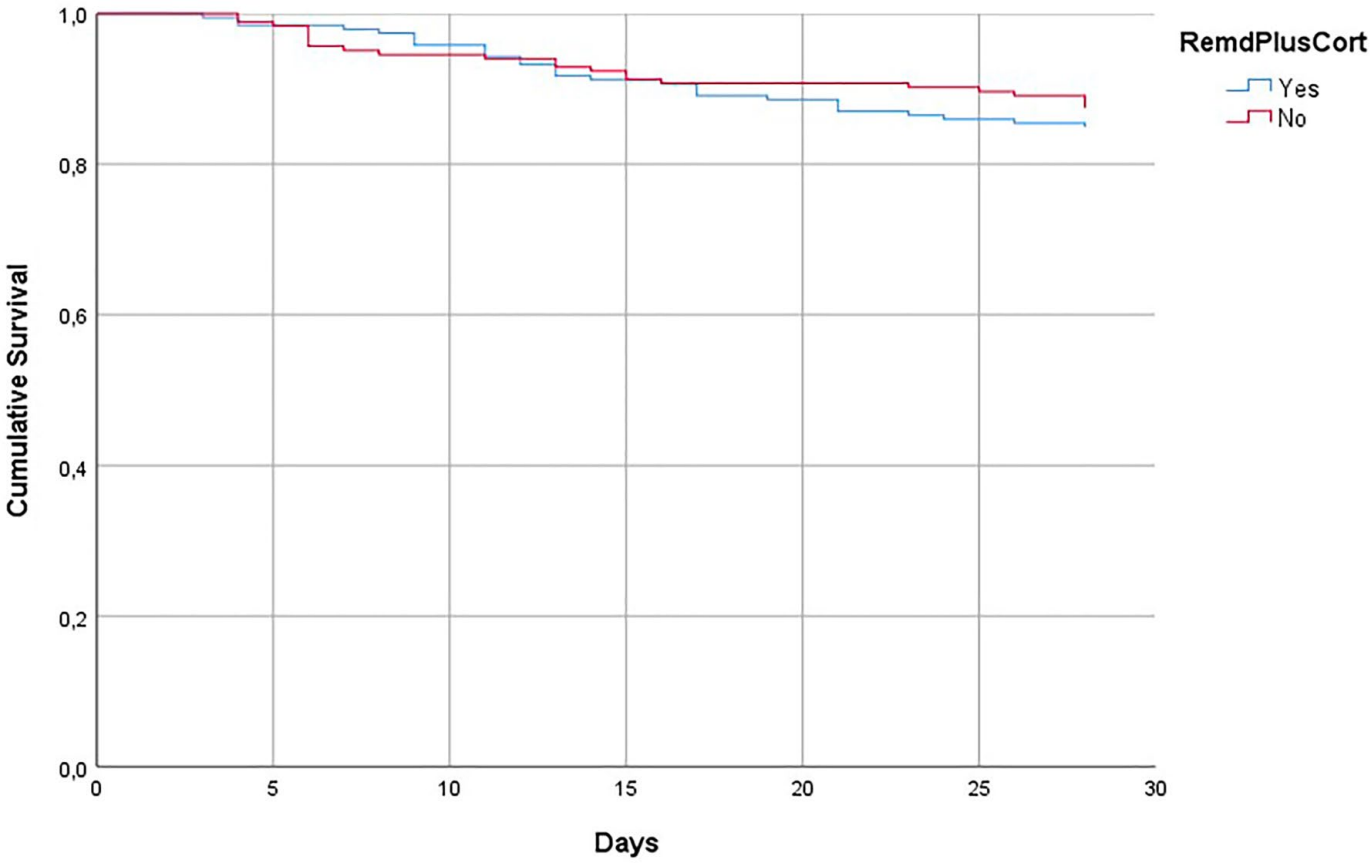
Kaplan–Meier Survival curves of patients treated with remdesivir and the association of remdesivir and corticosteroids adjusted for WHO Ordinal Scale severity and comorbidities.

Patients treated with RDV alone presented a lower, although not significant, mortality (12.5% vs. 15.2%, *p* = 0.44). The Kaplan–Meier survival curves at day 28 showed no difference between survivors and non-survivors (log-rank test of 0.477) between groups, even after adjustment for comorbidities or severity of patients evaluated by WHO Ordinal Scale [OS = 6: HR = 1.12 (95% CI 0.47–2.68; *p* = 0.78); OS = 7: HR = 1.66 (95% CI 0.5–5.4; *p* = 0.40] (Fig. 2).

Adverse events or severe adverse events other than death were identified in 20 patients (10.9%) in the RDV group and in 16 patients (8.4%) in the RDV plus corticosteroid group (*p* = 0.422) (Table 3).

**Table 3:** Adverse events and treatment group.

MedDRA system organ class		RDV	RDV + CORT
		n = 184	n = 190
Renal and urinary disorders	Acute kidney injury	9	5
Hepatobiliary disorders	Cholestasis	1	0
	Hyperbilirubinemia	0	1
Investigations	Increased transaminases	7	2
	Alanine aminotransferase ≥ 5 times the upper level of normal	3	7
	ALT elevation + conjugated bilirubin increase	2	1
	Gamma GT increase	1	0

## Discussion

In our study we found that the combination of corticosteroids with remdesivir did not improve the time to clinical recovery of patients with COVID-19 in comparison with RDV alone. Also, the hospital length of stay of patients treated with remdesivir alone was significantly lower than patients treated with both drugs.

Although the anti-inflammatory effect of corticosteroids could improve the hyperinflammatory state probably associated with COVID-19 pneumonia, its use is associated with several potential serious adverse effects. Such examples include: higher incidence of hospital-acquired infections, hyperglycemia, delirium or neuromuscular weakness that could compromise the recovery of patients and increase the length of hospital stay^7^. Nevertheless, the adverse effects of corticosteroids were not systematically evaluated in our study, which could explain our findings in the time to clinical recovery and the length of stay.

Corticosteroids emerged as a treatment of COVID-19 patients after the RECOVERY trial that showed significantly higher survival in patients under invasive mechanical ventilation and on supplemental oxygen receiving dexamethasone^8^. However, the results in several randomized controlled trials (RCT) have been sometimes controversial^8–11^. Tomazini et al. showed that dexamethasone had a mean 6.6 versus 4.0 ventilator-free days in controls (*P* = 0.04)^9^. But there was no significant difference in 28-day all-cause mortality (56.3 vs. 61.5%) or in other clinical endpoints. Similarly, in the REMAP-CAP COVID-19 RCT, hydrocortisone had no impact in hospital mortality rates (30%, 26% and 33% in the fixed-dose, shock-dependent and no hydrocortisone, respectively)^10^. More recently, Dupuis et al.^11^ found in a cohort of 303 COVID-19 patients that early corticosteroid treatment was not associated with patient survival. The various randomized studies presented very different mortalities of patients with similar severities, so it is difficult to consistently assess the impact of corticosteroids on the mortality of COVID-19 patients. Also, in the study by Jung et al., the treatment with corticosteroids in COVID-19 patients with 70 years and older was associated with increased mortality. In our study, there were no difference in the mortality rate of patients treated with RDV and the association of RDV with corticosteroids. On the contrary, patients treated with corticosteroids presents a slightly higher, although not significant, mortality^12^. It should be noted that patients treated with corticosteroids were more severe at admission in the evaluation of OS. When adjustments for severity were made in the evaluation of mortality, the tendencies to higher mortality remained. Also, no impact of comorbidities was observed in the outcome.

In other studies^3–5,13^, treatment with remdesivir has not been shown to improve mortality. In our study, the combination with corticosteroids did not improve mortality. Monotherapy with remdesivir was better in improving time to recovery and in reducing the length of hospital stay. In the pandemic context, these results could be very useful to release resources for the treatment of increasing numbers of infected patients. Also, in a recent study comparing baricitinib plus remdesivir and dexamethasone plus remdesivir, similar mechanical ventilation-free survival by day 29 was found. However, the corticosteroid arm was associated with significantly more adverse events, treatment-related adverse events, and severe or life-threatening adverse events^14^.

The current safety profile of remdesivir is still incomplete. Increasing evidence has demonstrated that COVID-19 is associated with multiple organ involvement including lung, liver, gastrointestinal tract, heart, and kidney. It is therefore complex to distinguish the underlying causes of adverse events during remdesivir treatment^15^. In our study, the incidence of adverse effects associated with RDV was similar to that observed in other studies^3–5,13^

Our study is not a randomized controlled study, and could therefore have a significant bias in the distribution of patients between both treatment groups. However, being a real-life study, results are reinforced with a robust structure and prospectively collected data.

Also, the homogeneity of the compared groups limits some potential biases on the analysed outcomes.

## Conclusion

In this study, patients treated with RDV alone had a shorter length of hospital stay. The use of corticosteroids as adjunctive therapy of RDV was not associated with improvement in mortality of COVID-19 patients. These results raise the question whether corticosteroids represent a real advantage in the treatment of patients with COVID-19 pneumonia. In near future, the results of other trials could help define the role of RDV and corticosteroids in the treatment of COVID-19 patients.

## Supplementary Information

Supplementary Information.

## Data Availability

The datasets used and/or analysed during the current study are available from the corresponding author on reasonable request.
