# Supplementary material for: Remdesivir and corticosteroids in the treatment of hospitalized COVID-19 patients
Source: Sci Rep. 2023 Mar 18;13:4482. doi: 10.1038/s41598-023-31544-5 (PMC10024012; doi:10.1038/s41598-023-31544-5)

Supplementary material

Figure. Non adjusted Kaplan-Meier Survival curves of patients treated with remdesivir and the association of remdesivir and corticosteroids

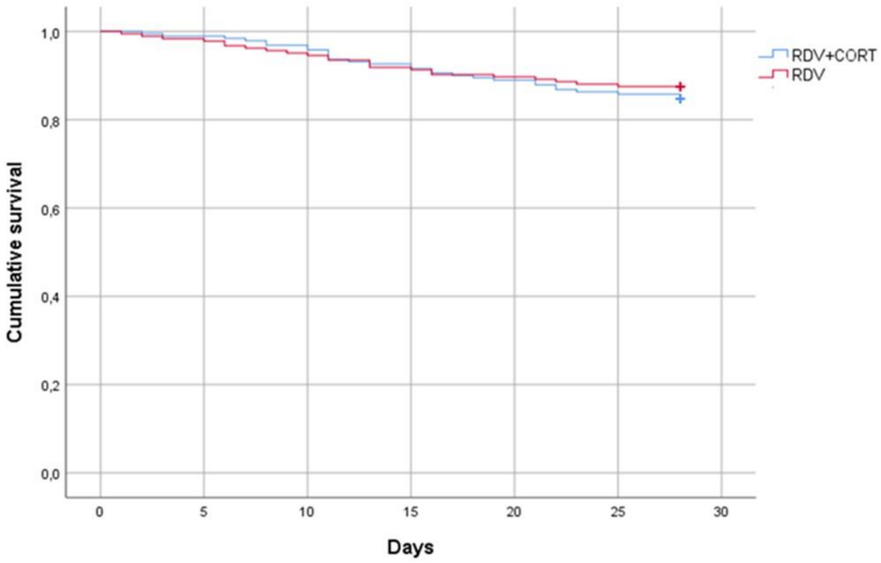

Supplement: Supplementary file 1 — Supplementary Information. [file 41598_2023_31544_MOESM1_ESM.pdf]
